# MRgFUS new applications in musculoskeletal pathology: a miscellaneous case review

**DOI:** 10.1186/2050-5736-3-S1-O46

**Published:** 2015-06-30

**Authors:** Alberto Bazzocchi, Alessandro Napoli, Giacomo Filonzi, Maurizio Busacca, Emanuela Palmerini, Stefano Ferrari, Carlo Catalano, Ugo Albisinni

**Affiliations:** 1 The “Rizzoli” Orthopaedic Institute, Bologna, Italy; 2 University of Rome – Sapienza, Rome, Italy

## Background/introduction

Magnetic resonance guided focused ultrasound surgery (MRgFUS) of the musculoskeletal system achieved significant results in the treatment of painful bone metastases, and recently showed promise for benign bone lesions (e.g. osteoid osteoma) and osteoarthritis (e.g. facet joint syndrome and knee osteoarthritis). MRgFUS works on the main basis of pain relief and tumor control / killing. In this case review five patients affected by different diseases not commonly treated by MRgFUS are presented.

## Methods

(A) A patient suffering from femuroacetabular impingement developed a small focal lesion in the anterosuperior profile of the femoral neck (herniation pit). The lesion was harshly painful at baseline (visual analogue scale score – VAS 9), with severe mobility impairment. Conservative treatments failed and the patient was in list for arthroplasty. (B and C) In 2 patients, the targeted lesion was a small (about 1 cm) subcortical alteration of the posterosuperior profile of the femoral neck, diagnosed as a focal degenerative change caused by posterior impingement syndrome. At baseline patients suffered from pain (VAS 9 and 8, respectively) and relevant mobility impairment like in case A; both patients started with symptoms several months before and they were unsuccessfully treated by drugs and surgery (arthroscopic approach – male patient). (D) A patient was found with a 1-cm sclerotic alteration of the 8th right rib, dimensionally stable at 6-month imaging follow-up; scintigraphy and FDG positron emission tomography were both negative, with no nidus or other signs detected by CT or perfusion MR. No history of oncological disorders. The lesion was painful (VAS 9), and previous treatments (systemic drugs, local injections) failed in controlling pain. We decided to proceed to MRgFUS without histological diagnosis (fibrous dysplasia, osteoid osteoma or osteitis were the most likely hypotheses). (E) Another patient with aggressive fibromatosis presented with a 10-cm mass extended from the left popliteal fossa to the leg; the patient previously underwent several surgical sessions, radiation therapy and pharmacological treatment throughout two decades, particularly over the site of the presented fibroma (large scars, venous varicosity and incontinence, and arterial bypass). The patient suffered from relapses and also infective and vascular complications. All patients were examined clinically and by imaging, before treatment (ExAblate 2100, InSightec) and up to 12 months of follow-up (1-, 3-, 6- and 12-month checks).

## Results and conclusions

Patients with focal degenerative diseases (A, B, and C) showed an excellent response to treatment: VAS dropped to 0 after 1 month and persisted to 0 in all the subsequent follow-up controls (12 months), with significant improvement in the mobility of the affected hip. The patient (D) with rib lesion of unknown origin had only a partial improvement (VAS from 9 to 6 after 1 month, the only check reached).

The patient affected by fibromatosis showed VAS flattening to 0 (1-month check) with improvement of the perceived quality of life. The lesion showed dimensional reduction and no enhancement at contrast media injection from the 3-month control (6 months of follow-up). Several and different applications of MRgFUS in musculoskeletal disorders are coming, to find an effective solution or to minimize invasivity and avoid or postpone any major intervention.

